# Epstein–Barr virus-associated posttransplant lymphoproliferative disorder involving the central nervous system following autologous hematopoietic stem cell transplantation for neuroblastoma

**DOI:** 10.1186/2193-1801-3-582

**Published:** 2014-10-06

**Authors:** Hitoshi Sano, Masanobu Fujimoto, Keisuke Okuno, Jun-ichi Ueyama, Shuichi Takano, Kazuhiko Hayashi, Susumu Kanzaki

**Affiliations:** Division of Pediatrics and Perinatology, Faculty of Medicine, University of Tottori, 36-1 Nishi-cho, Yonago, 683-8504 Japan; Division of Molecular Pathology, Faculty of Medicine, University of Tottori, Yonago, Japan; Division of Pediatric Surgery, Faculty of Medicine, University of Tottori, Yonago, Japan

**Keywords:** Posttransplant lymphoproliferative disorder, Epstein-Barr virus, Central nervous system, Autologous hematopoietic stem cell transplantation, Neuroblastoma

## Abstract

**Introduction:**

Posttransplant lymphoproliferative disorder (PTLD) is a serious complication following solid organ or hematopoietic stem cell transplantation (HSCT). Although extranodal involvement of PTLD is common, its isolated involvement in the central nervous system (CNS) is extremely rare. To date, primary CNS-PTLD has been reported in 13 patients who underwent allogeneic HSCT, but no cases have been reported in autologous HSCT recipients.

**Case Description:**

Herein, we report the first report of a patient with neuroblastoma that progressed to CNS-PTLD after autologous peripheral blood stem cell transplantation (auto-PBSCT). A 27-month-old boy with stage IV neuroblastoma of the left adrenal gland received auto-PBSCT after intensive chemotherapy, tumor resection, and radiation of tumor bed and regional lymph node. An intracranial tumor in his left parietal lobe was detected by magnetic resonance imaging 99 days posttransplantation, and the tumor was completely resected. The histological diagnosis of the intracranial tumor was diffuse large B-cell lymphoma with latency type III Epstein-Barr virus infection. The patient has maintained tumor free status 3 years after auto-PBSCT.

**Discussion and Evaluation:**

Given the rarity of CNS-PTLD, there is no consensus on the optimal treatment. Historically, the outcome of CNS-PTLD has been very poor. However, our patient remains free from PTLD after only total resection. The prognosis for PTLD following auto-HSCT may depend upon the underlying malignancy, immune state, EBV immune status, and treatments.

**Conclusions:**

The outcome of PTLD following auto-HSCT is not necessarily poor prognosis. Further research is required to establish the optimal treatment strategy for CNS-PTLD.

## Background

Posttransplant lymphoproliferative disorder (PTLD) is one of the possible life-threatening complications following solid organ or hematopoietic stem cell transplantation (HSCT). The incidence of PTLD is dependent on the type of transplanted organ, the type and intensity of immunosuppression, and the patient’s immune status to Epstein–Barr virus (EBV) (Lim et al., 2006; Gottschalk et al., 2005). Following allogeneic HSCT (allo-HSCT), the risk factors associated with PTLD are donor T-cell depletion, the use of anti-thymocyte globulin (ATG), unrelated or human leukocyte antigen (HLA)-mismatched grafts, and acute or chronic graft-versus-host disease (Landgren et al., 2009). PTLD typically occurs after prolonged and profound immunosuppression, which decreases the number of EBV-specific cytotoxic T-lymphocytes, resulting in uncontrolled EBV-induced B-lymphocyte proliferation (Penn, 1994).

Extranodal involvement is common in PTLD, but the isolated involvement of the central nervous system (CNS) is extremely rare. CNS involvement occurs in approximately 10–20% of solid organ transplant recipients (Penn and Porat, 1995; Buell et al., 2005; Cavaliere et al., 2010). To date, primary CNS-PTLD (PCNS-PTLD) has been reported in 13 patients who underwent allo-HSCT, but no cases have been reported in autologous HSCT (auto-HSCT) recipients (Lieberman et al., 2012; Eckrich et al., 2012).

Given the rarity of PCNS-PTLD, there is no consensus about the optimal treatment. In solid organ transplant recipients, the median time from transplantation to PCNS-PTLD is 4.4 years (Cavaliere et al., 2010). Conversely, the incidence of PTLD peaks within the first year following allo-HSCT (Landgren et al., 2009).

Herein, we report the first case of PCNS-PTLD in an infant following auto-HSCT for neuroblastoma.

### Case description

**Figure 1:**
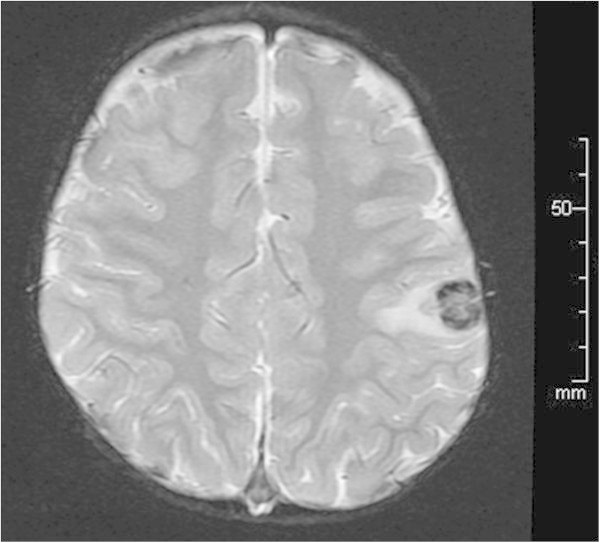
**T2-weighted magnetic resonance imaging revealed an intracranial tumor in the left parietal lobe, accompanied by extensive peritumoral edema.**

**Figure 2:**
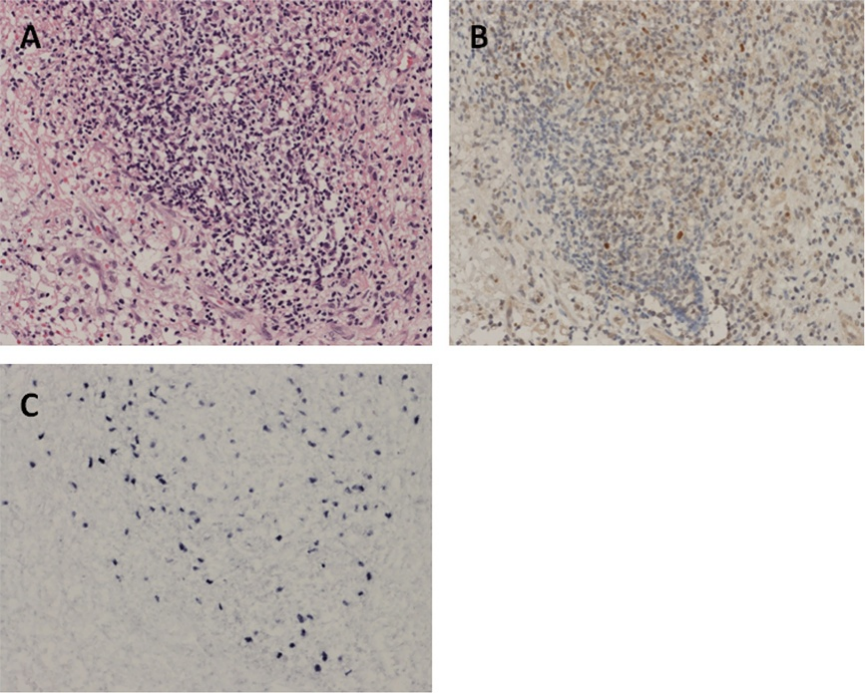
**Histological analysis of the brain tumor demonstrated an abnormal infiltration of pleomorphic lymphoid cells with a dominant component of large lymphoid cells (A; hematoxylin-eosin stain).** Nuclear expressions for Epstein–Barr virus (EBV) nuclear antigen 2 (EBNA-2) and EBV-encoded RNA 1 (EBER1) were detected with immunohistochemistry **(B)** and in situ hybridization **(C)**, respectively. The pathological diagnosis was diffuse large B-cell lymphoma with latency type III, EBV-infection.

A 20-month-old boy was admitted to our hospital for stage IV neuroblastoma involving the left adrenal gland, left neck lymph nodes, and bone marrow of the right femur. No abnormal lesions were detected on a computed tomography scan of the brain. The patient underwent 5 cycles of chemotherapy consisting of cyclophosphamide (1,200 mg•m - 2•dose - 1 × 2 days (excluding first cycle; 1 day) ), vincristine (1.5 mg•m - 2•dose - 1 × 1 day), pirarubicin (40 mg•m - 2•dose - 1 × 1 day), and cisplatin (20 mg•m - 2•dose - 1 × 5 days). After the third and fourth cycles of the chemotherapy, peripheral blood stem cells were harvested, and the primary tumor (i.e., the left adrenal gland) was completely resected. After 1 more cycle of chemotherapy, when the patient was 27 months old, autologous peripheral blood stem cell transplantation (auto-PBSCT) was performed. The conditioning regimen consisted of carboplatin (400 mg•m - 2•dose - 1 × 4 days), etoposide (200 mg•m - 2•dose - 1 × 4 days), and melphalan (100 mg•m - 2•dose - 1 × 2 days). The patient received an infusion of 1.76 × 10^6^ CD34+ cells/kg of autologous unmanipulated peripheral blood stem cells, and achieved neutrophil engraftment on day 12. Starting on day 49 after auto-PBSCT, the patient received abdominal involved field radiation of 19.8 Gy in 11 fractions. After treatment, the patient vomited 2 or 3 times per week, usually in the morning. The reasons for the vomiting were investigated. An upper gastrointestinal series was performed, and gastroesophageal reflux disease was detected. The patient was administered famotidine, and the vomiting ceased. Subsequently, magnetic resonance imaging on day 99 after auto-PBSCT revealed an intracranial tumor in the left parietal lobe, and the tumor was accompanied by extensive peritumoral edema (Figure 1). The tumor was completely resected. The resected tumor contained a lymphoid infiltrate composed of many large atypical lymphocytes with prominent nuclei. Immunohistochemical analysis demonstrated that the large lymphocytes exhibited a B-cell phenotype, with positive immunoreactivity for CD20, CD79a, and MUM-1, and negative staining for CD3 and CD45RO. Immunohistochemistry showed expression of EBV nuclear antigen-2 (Figure 2B), and latent membrane protein 1 in the lymphocytes, and *in situ* hybridization indicated nuclear expression of EBV-encoded RNA-1 (Figure 2C). The pathological diagnosis was diffuse large B-cell lymphoma with latent type III EBV-infection. A final diagnosis of diffuse large B-cell lymphoma with EBV associated PTLD was made. EBV statuses were examined after diagnosis of PTLD. EBV-EA IgG antibody and EBV-VCA IgM were negative, but EBV-VCA IgG and EBV-EBNA IgG were positive. EBV-DNA in blood has not been detected. And, absolute CD4, CD8, and lymphocyte count were 174/μl, 470/μl, and 1,460/μl, respectively (CD4/CD8 ratio; 0.37). The patient has no neurological symptoms and remains free from neuroblastoma and EBV-associated PTLD 3 years after auto-PBSCT.

## Discussion

PCNS-PTLD was first reported by Schneck and Penn in 1970 (Schneck and Penn, 1970). Since then, the frequency of transplantation has increased, and improvements in supportive care and immunosuppression have been made. CNS-PTLD is a rare but important complication in graft patients, particularly in HSCT recipients. It has been reported that the incidence of PTLD after allo-HSCT is 0.47–1% (Landgren et al., 2009; Curtis et al., 1999). Lieberman et al. have reviewed 13 patients with PCNS-PTLD following allo-HSCT (Lieberman et al., 2012), and Eckrich et al. have reviewed the incidence of PTLD following pediatric auto-HSCT (Eckrich et al., 2012). In their report, Eckrich et al. indicate that the diseases underlying PTLD were neuroblastoma (5 cases), retinoblastoma (1 case), nodular sclerosis Hodgkin’s lymphoma (1 case), and unspecified disease (1 case). Our case of infantile PCNS-PTLD after auto-HSCT is extremely rare, and no similar case has been reported.

Landgren et al. have indicated that the risk factors associated with PTLD following allo-HSCT are T-cell depletion of the donor marrow, ATG use, and unrelated or HLA mismatched grafts (Landgren et al., 2009). However, because of the scarcity of the disease, the risk factors for PTLD following auto-HSCT remain undefined. EBV infection is a common risk factor for PTLD. In our case, histopathological analysis revealed that EBV infection was the etiology of PTLD, although it was unclear when the patient was infected with EBV. The prognosis for PTLD following auto-HSCT may depend upon the underlying malignancy, immune state, EBV immune status, and treatments.

Given the rarity of PCNS-PTLD, there is no consensus on the optimal treatment. Historically, the outcome of PCNS-PTLD has been very poor. Buell et al. have reported that radiotherapy is the only effective therapy (Buell et al. 2005). Chemotherapies, including rituximab, are not effective because of the blood-brain barrier; the level of rituximab in the cerebrospinal fluid has been reported to be approximately 0.1% of serum levels (Rubenstein et al., 2003). In their report, Eckrich et al. indicate that 4 of 7 patients with PTLD after auto-HSCT received rituximab (Eckrich et al. 2012). All these patients underwent a successful resolution of their PTLD, but 3 died from their underlying malignancies. Recently, some trials have been conducted in which intrathecal (van de Glind et al., 2008) or mass administration (Traum et al, 2006) of rituximab was performed. However, although these treatments may be promising for patients with CNS-PTLD, further research is required to establish the optimal treatment strategy.

## Conclusions

The outcome of PTLD following auto-HSCT is not necessarily poor prognosis. Further research is required to establish the optimal treatment strategy for CNS-PTLD.

## Abbreviations

PTLD: Posttransplant lymphoproliferative disorder
HSCT: Hematopoietic stem cell transplantation
CNS: Central nervous system
auto-PBSCT: Autologous peripheral blood stem cell transplantation
EBV: Epstein–Barr virus
ATG: Anti-thymocyte globulin
HLA: Human leukocyte antigen
allo-HSCT: Allogeneic hematopoietic stem cell transplantation
PCNS-PTLD: Primary central nervous system posttransplant lymphoproliferative disorder
auto-HSCT: Autologous hematopoietic stem cell transplantation.
